# Epigenetic Remodeling through Downregulation of Polycomb Repressive Complex 2 Mediates Chemotherapy Resistance in Testicular Germ Cell Tumors

**DOI:** 10.3390/cancers11060796

**Published:** 2019-06-08

**Authors:** Ratnakar Singh, Zeeshan Fazal, Andrea K. Corbet, Emmanuel Bikorimana, Jennifer C. Rodriguez, Ema M. Khan, Khadeeja Shahid, Sarah J. Freemantle, Michael J. Spinella

**Affiliations:** 1 Department of Comparative Biosciences, University of Illinois at Urbana-Champaign, Urbana, IL 61801, USA; rsingh02@illinois.edu (R.S.); fazal2@illinois.edu (Z.F.); acorbet2@illinois.edu (A.K.C.); ebikorim@illinois.edu (E.B.); jrodr53@illinois.edu (J.C.R.); ekhan4@illinois.edu (E.M.K.); kshahi4@illinois.edu (K.S.); sarahf@illinois.edu (S.J.F.); 2 Carle Illinois College of Medicine and Cancer Center of Illinois, University of Illinois at Urbana-Champaign, Urbana, IL 61801, USA

**Keywords:** cisplatin, testicular cancer, polycomb repressive complex, histone methylation, H3K273me, epigenetics

## Abstract

A greater understanding of the hypersensitivity and curability of testicular germ cell tumors (TGCTs) has the potential to inform strategies to sensitize other solid tumors to conventional chemotherapies. The mechanisms of cisplatin hypersensitivity and resistance in embryonal carcinoma (EC), the stem cells of TGCTs, remain largely undefined. To study the mechanisms of cisplatin resistance we generated a large panel of independently derived, acquired resistant clones from three distinct parental EC models employing a protocol designed to match standard of care regimens of TGCT patients. Transcriptomics revealed highly significant expression changes shared between resistant cells regardless of their parental origin. This was dominated by a highly significant enrichment of genes normally repressed by H3K27 methylation and the polycomb repressive complex 2 (PRC2) which correlated with a substantial decrease in global H3K27me3, H2AK119 ubiquitination, and expression of BMI1. Importantly, repression of H3K27 methylation with the EZH2 inhibitor GSK-126 conferred cisplatin resistance to parental cells while induction of H3K27 methylation with the histone lysine demethylase inhibitor GSK-J4 resulted in increased cisplatin sensitivity to resistant cells. A gene signature based on H3K27me gene enrichment was associated with an increased rate of recurrent/progressive disease in testicular cancer patients. Our data indicates that repression of H3K27 methylation is a mechanism of cisplatin acquired resistance in TGCTs and that restoration of PRC2 complex function is a viable approach to overcome treatment failure.

## 1. Introduction

Testicular germ cell tumors (TGCTs) represent the most common carcinoma of young men between the age of 15–35 [1]. The TGCTs are thought to arise from the transformation of primordial germ cells (PGCs) [2] and are divided into two major histological types, seminomas and non-seminomas. The non-seminomas are further divided into embryonal carcinoma (EC), teratoma, yolk sac tumor, and choriocarcinoma. Pluripotent ECs represent the stem cells of non-seminomas and differentiate into mature non-seminoma subtypes [2].

Cisplatin is the first-line therapy for a number of cancers including TGCTs. Unlike most solid tumors, advanced TGCTs can be cured at a high rate with conventional chemotherapy that features cisplatin in combination with bleomycin, etoposide or vinblastine [3,4]. Approximately 80% of TGCT patients are cured of their disease with cisplatin-based combination chemotherapy even when the disease is highly metastatic [5,6,7]. Further, there are patients who initially respond to therapy but undergo late relapse that are essentially incurable [8]. Therapies to treat these cisplatin resistant populations is a major unmet clinical need. TGCTs have an intrinsic hypersensitivity to cisplatin-induced cell death [9,10]. However, the basis of this hypersensitivity and mechanisms to account for chemotherapy resistance remains elusive.

A greater understanding of the hypersensitivity and curability of TGCTs has the potential to inform strategies to sensitize other solid tumors to conventional chemotherapies. This is highlighted by evidence suggesting that alterations in traditional pathways of inherent or acquired cisplatin resistance, common to other tumors, do not explain the exquisite sensitivity of TGCTs to cisplatin therapy [11]. In comparison to other tumors, TGCTs possess unique epigenetic states due to the origins from primordial germ cells, including distinct patterns of DNA methylation and repressive and active histone modifications associated with pluripotent states [12]. Aberrant epigenetics are likely a major driving force in TGCT etiology and progression, since these tumors have a relative paucity of genetic alterations in cancer drivers [13,14,15]. However, the relationship between TGCT epigenetics and curability and chemoresistance is largely uncharacterized and has been limited to a few studies related to gene specific DNA methylation [10,12].

In the current study, we employed unique cisplatin resistant cell models and de novo transcriptional profiling approaches that unexpectedly uncovered an important role for polycomb in TGCT sensitivity and resistance to cisplatin. A highly significant upregulation of genes normally repressed by H3K27 methylation and the polycomb repressive complex 2 (PRC2) was noted in cisplatin resistant TGCT cells regardless of their parental origin which correlated with a substantial decrease in global H3K27me3, H2AK119 ubiquitination, and expression of BMI1. Importantly, repression of H3K27 methylation conferred cisplatin resistance to parental cells, while induction of H3K27 methylation resulted in increased cisplatin sensitivity. A gene signature based on H3K27me gene enrichment predicted an increased rate of recurrent/progressive disease in testicular cancer patients. Our findings are the first to implicate histone methylation with chemosensitivity of TGCTs. Furthermore, we provide evidence that repression of H3K27 methylation is a mechanism of cisplatin acquired resistance in TGCTs and suggest that drugs targeting PRC2 function are a viable approach to overcome treatment failure in TGCT patients and to potentially sensitize other solid tumors to chemotherapy.

## 2. Results

### 2.1. Acquired Cisplatin Resistance in Testicular Cancer Cells Is Associated with Enhanced Expression of Genes Normally Repressed by H3K27 Methylation

**Figure 1:**
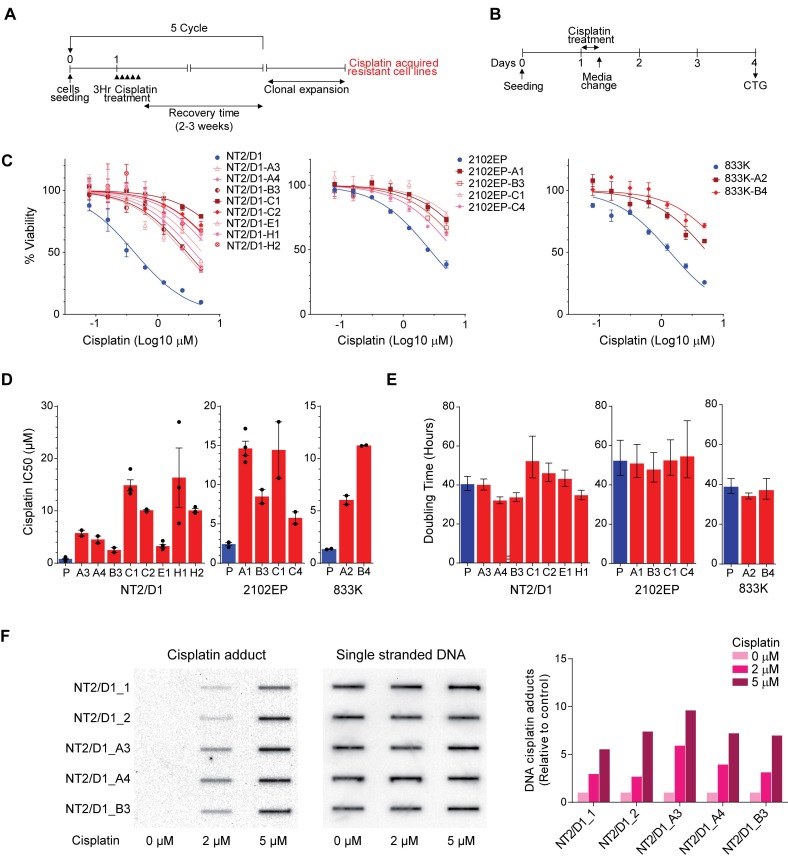
Development of cisplatin acquired resistant TGCT cell lines. (**A**) Three human testicular cancer derived embryonal carcinoma cell lines NT2/D1, 833K, and 2102EP were exposed to stepwise dosages of cisplatin starting at 0.5 μM for 3 h for 5 consecutive days and then allowed to recover for 2–3 weeks per cycle for 5 cycles. Each solid triangle represents 3 h of cisplatin treatment for 5 consecutive days. At the end of 5th cycle cisplatin-refractory cells were cloned. (**B**) Schematic of cisplatin treatment for cell survival and viability experiments. Parental and acquired resistant TGCT cells were treated with cisplatin for 6 h and assayed for survival 3 days later. CTG = CellTiter-Glo. (**C**) Cisplatin selected cell lines are stably resistant to cisplatin. Cell survival and viability was measured using CellTiter-Glo in parental or acquired resistant TGCT cells treated with indicated cisplatin doses. (**D**) Cisplatin IC50 values for paternal and cisplatin resistant cells were estimated from the best-fit dose-response model. (**E**) Cisplatin resistant cell lines have similar doubling times as compared to parental cells. Doubling time were calculated by an exponential growth curve equation from viable cell numbers measured for 5 consecutive days using the CellTiter-Glo assay. All data represent mean ± standard error of the mean. (**F**) Cisplatin resistant lines have similar cisplatin adduct formation. Representative immunoblot of cells treated with indicated dosages of cisplatin for 3 h. DNA was isolated for immunoblot analysis using platinum-GG (Pt-GG) adduct antibody. An anti-single stranded DNA (ssDNA) antibody served as the loading control.

In order to elucidate mechanisms of acquired cisplatin resistance in testicular cancer, we generated a series of clonal cisplatin resistant cell lines from three randomly chosen parental embryonal carcinoma cell lines that are hypersensitive to cisplatin, NT2/D1, 833K, and 2102EP. The protocol chosen was designed to mimic how cisplatin-based therapies are administered in the clinic, namely as daily (day one to five) 20 mg/m^2^ IV administrations with repeated cycles every 21 days. This dosage has been reported to achieve a 1–4 µM peak cisplatin concentration that declines rapidly within 2 h after infusion [16]. Patients who fail this frontline cisplatin therapy often receive higher doses of the cisplatin analog, carboplatin [6]. Hence, cells were treated with 0.5 µM cisplatin for 3 h on 5 consecutive days and then allowed to recover for two to three weeks (Figure 1A). This cycle was repeated 5 times with escalating doses of cisplatin with a final selection at 10 µM cisplatin. For each parental line, resistant clones were derived from independently treated batches of cells as described in Material and Methods.

In total eight, four and two independently acquired resistant clones of parental NT2/D1, 833K, and 2102EP embryonal carcinoma cells were generated, respectively (Supplementary Table S1). The characterization of cell viability (as described in Figure 1B) confirmed stable cisplatin resistance ranging from three- to 30-fold as compared with parental cell lines (Figure 1C,D and Supplementary Table S1). The resistant lines had similar doubling times as the parental cells (Figure 1E) and each cell line was stably resistant to cisplatin as resistance was maintained for extended passages (up to 4 months) in cisplatin-free media. Cisplatin adduct formation was similar in the parental and resistant cells after acute 3-hour exposure to cisplatin, suggesting that the resistance was not due to decreased cisplatin uptake (Figure 1F).

**Figure 2:**
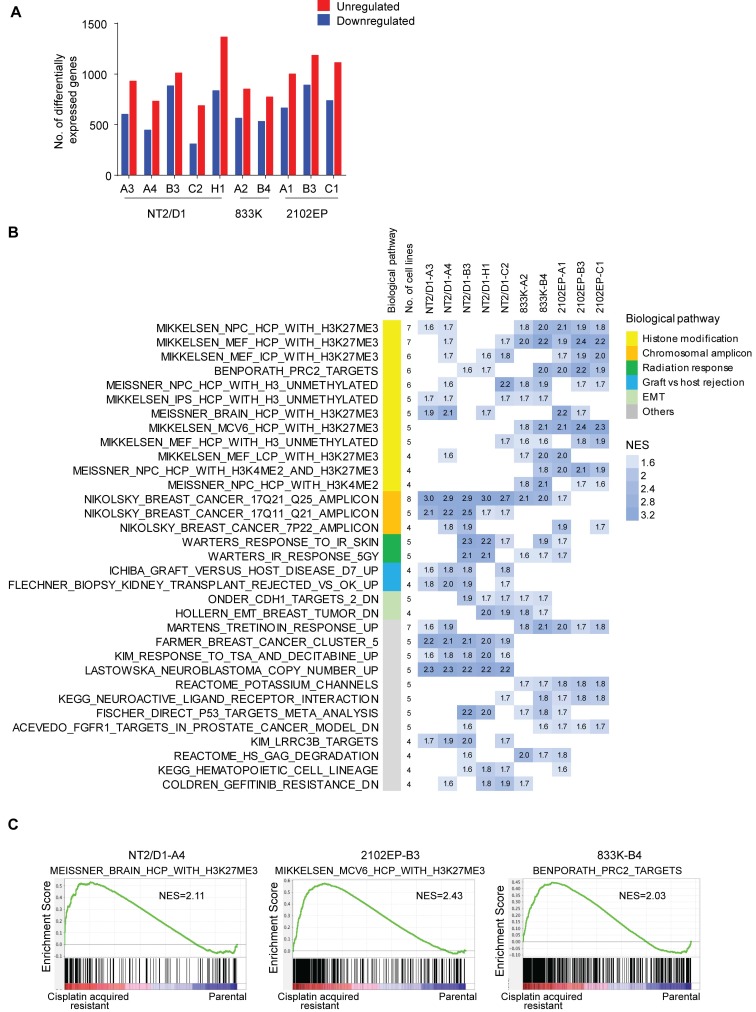
Cisplatin resistance in testicular cancer cells is associated with enhanced expression of genes normally repressed by H3K27 methylation. (**A**) Number of genes upregulated and downregulated in cisplatin resistant cells lines as compared to respective parental control cells. A 2-fold cut off and FDR < 0.01 was used. Average of biological triplicate determinations. (**B**) Cisplatin resistant cells are enriched for gene sets associated with H3K27 methylation and PRC2 targets. Gene set enrichment analysis (GSEA) indicating all gene sets that were enriched from 4762 curated gene gets from the MSigDB C2 collection in cisplatin resistant cells as compared with respective parental cells. All gene sets that have a normalized enrichment score (NES) of 1.6 or greater and are enriched in four or more resistant cell lines are included. Numbers and gradient of blue color represent NES. (**C**) Representative gene set enrichment plots. H3K27me3, histone 3 K27 trimethylation; PRC2, polycomb repressive complex 2; NES, normalized enrichment score.

In order to explore mechanisms of cisplatin resistance in testicular cancer cells, RNA-seq transcriptomic profiling and differential gene expression analysis was performed on 10 randomly chosen resistant lines. In total, 800–1500 genes were differentially expressed at least 2-fold with an FDR < 0.01 between cisplatin resistant cells and respective parental cells (Figure 2A and Supplementary Figure S1). The gene set enrichment analysis (GSEA) revealed that the cisplatin resistant cells, as compared with their respective parental cells, exhibited dramatic upregulation in genes associated with H3K27 methylation and polycomb repressive complex 2 (PRC2) pathways (Figure 2B,C and Supplementary Table S2). The enrichment in genes normally repressed by PRC2 was observed across the resistant cell lines regardless of the parental cell of origin and was the most consistent enrichment noted with the 10 lines having a range of one to 10 distinct PRC2-related gene sets with a normalized enrichment score (NES) of 1.6 or greater (median number of gene sets per line = 7) (Figure 2B and Supplemental Table S2). We also performed targeted GSEA analysis using four distinct polycomb-related gene sets from human ES cells which are EED targets, SUZ12_TARGETs, H3K27_BOUND, and PRC2_TARGETS. The majority of the cisplatin resistant cells lines showed highly significant enrichment for each of these gene sets (Supplementary Figure S2).

Another notable enrichment was the genes on chromosomal region 17q21-25, suggesting that this region may have been amplified in a subset of the resistant lines. However, we were unable to find evidence of focal amplification of this region in testicular cancer patients from the TCGA database. Other gene sets related to graft vs. host rejection, radiation response, and epithelial to mesenchymal transition were also enriched but with a lesser frequency (Figure 2B and Supplementary Table S2). Consistent with the premise that cisplatin resistant cells repress PRC2 signaling, GSEA also revealed that resistant cells decreased expression of genes that were downregulated when PRC2 component SUZ12 was deleted in mouse ES cells [17] (Supplementary Figure S3).

### 2.2. Cisplatin Resistance in Testicular Cancer Cells Is Associated with Decreased H3K27 Methylation, H2A-K119 Ubiquitination and Decreased Expression of BMI1 and EZH2.

**Figure 3:**
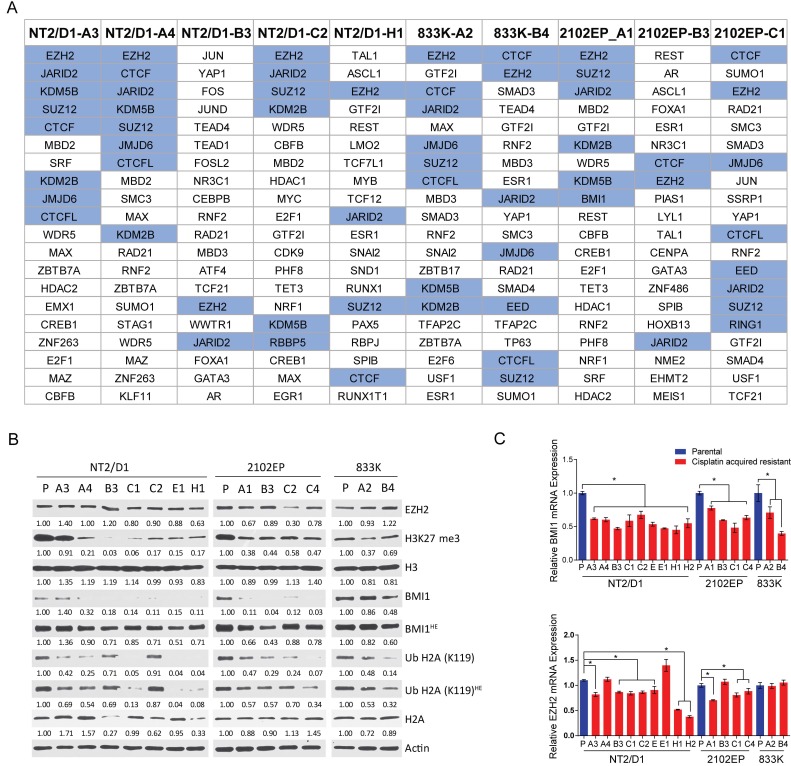
Cisplatin resistance in testicular cancer cells is associated with decreased H3K27 methylation, H2A-K119 ubiquitination, and decreased expression of BMI1 and EZH2. (**A**) Top 20 transcription factors predicted to bind to promotors of genes upregulated in cisplatin resistant cells as compared with parental cells using binding analysis for the regulation of transcription (BART). The transcription factors are arranged based on relative rank. The PRC1/2-related transcription factors are highlighted in blue. (**B**) Cisplatin resistant cells have decreased H3K27me3 and H2A-K119-ubiqutination as compared with parental cells and have reduced expression of BMI1. Immunoblot analysis of indicated cell lines with antibodies recognizing H3K27-me3, H2A-K119-Ub, BMI1, and EZH2. Actin, H3, and H2A expression served as loading control. HE = higher exposure. (**C**) Real-time PCR analysis of mRNA expression of EZH2 and BMI1 in parental and cisplatin acquired resistant TGCT cells. Samples were independent from those used in RNA-seq analysis. Data is mean ± standard error of the mean. * = *p* > 0.05.

BART (binding analysis for regulation of transcription) is an analysis that predicts upstream transcription factor binding from gene expression data based on promoter/enhancer ChIP-seq data [18]. The BART analysis predicts functional transcription factors that regulate a query gene set based on more than 6000 existing ChIP-seq datasets for over 400 factors in human and mouse cells. The BART analysis of genes upregulated in resistant cells, as compared with parental controls, strongly predicted polycomb complex component binding in all of the resistant cells regardless of their parental origin. The number of distinct PRC1/PRC2 related components contained in the top 20 predicted transcription factor binders for each line ranged from two to eight (median six) and included EZH2, JARID2, SUZ12, EED, KDM2B, KDM5B, and JMJD4 (Figure 3A and Supplementary Table S3). These results further support that the main transcriptional change in the cisplatin resistant cells is mediated by polycomb complexes. We also considered whether there was a common set of genes upregulated in the resistant cell lines as compared with each matching parental cell lines. A total of 89 genes were found to be commonly upregulated in at least 80% of the 10 resistant cells with a >1.5 fold-change and *p* value < 0.05. The BART analysis showed that five of the top eight predicted transcription factors regulating these 89 genes were also in the polycomb pathway (Supplementary Table S4).

Consistent with the upregulation in expression of genes normally repressed by PRC1/PRC2, the PRC2-mediated repressive mark H3K27me3 was consistently downregulated in the resistant cells as was the PRC1 repressive mark H2A-K119Ub (Figure 3B). In order to begin to assess the mechanism of PRC1/2 alteration in the resistant cells, expression of a number of PRC1/2 components was assessed. On the basis of the RNA-seq data, a decreased expression of PRC2 complex genes RBBP4, RBBP7, PHF2, and YY1 was noted in seven to 10 of the lines as compared with their parental control lines. Similarly, a significantly decreased expression of PRC1 complex genes BMI1, PCGF6, PCGF1, RYBP, CBX6, SCMH1, and L3MBTL1 was noted in seven to 10 of the lines as compared with parental cell lines.

On the basis of the Western and RT-PCR analysis, BMI1 levels were consistently decreased in resistant cells as compared with parental cells, and EZH2 was repressed in a smaller subset of cells (Figure 3B,C). This data suggest some of the individual cell lines may have achieved polycomb pathway repression by decreased expression of distinct PRC1/2 components, and also suggest some of the lines may have downregulated H3K27me3 or H2A-K119Ub by mechanisms other than downregulation of BMI1 or EZH2 expression.

### 2.3. Inhibition of H3K27 Methyltransferase EZH2 Results in Cisplatin Resistance of Testicular Cancer Cells and Inhibition of H3K27 Demethylase JMJD3 Sensitizes Testicular Cancer Cells to Cisplatin

**Figure 4:**
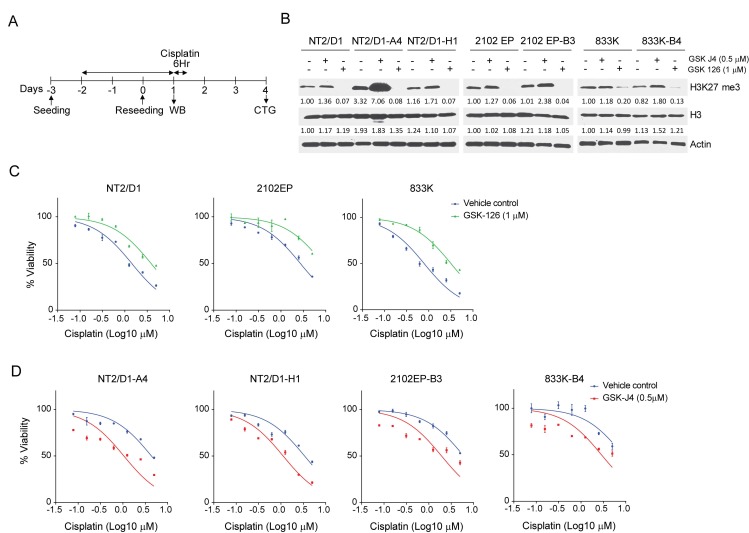
Inhibition of H3K27 methylation mediates cisplatin resistance in testicular cancer cells and inhibition of H3K27 demethylation sensitizes testicular cancer cells to cisplatin. (**A**) Schematic of protocol for pretreatment with H3K27 methyltransferase or demethylase inhibitors followed by cisplatin survival and viability assay in parental and cisplatin resistant TGCT cells. Parental and cisplatin resistant cells were treated with H3K27 methyltransferase inhibitor, GSK126, (1 µM) or demethylase inhibitor, GSK J4, (0.5 µM) for 72 h. After pretreatment cells were either used for immunoblotting or cisplatin viability assay. CTG = CellTiter-Glo. (**B**) Treatment with GSK126 or GSKJ4 alters H3K27me3 levels. Immunoblot analysis for expression of H3K27me. Actin and H3 expression served as loading controls. (**C**) Pretreatment of TGCT parental cells NT2/D1, 2102EP, and 833K with the EZH2 H3K27 methyltransferase inhibitor, GSKJ4, results in cisplatin resistance. (**D**) Pretreatment of cisplatin resistant cells NT2/D1-A4, NT2/D1-H1, 2102EP-B3, and 833K-B4 with the JMJD3 histone demethylase inhibitor, GSKJ4, results in increased cisplatin sensitivity. All data represent mean ± standard error of the mean.

To test the hypothesis that PRC1/2 mediated epigenetic changes are involved in cisplatin acquired resistance, cisplatin sensitive parental cells, NT2/D1, 2102EP, and 833K were pretreated with the specific EZH2 H3K27 methyltransfase inhibitor, GSK126, for three days at a dosage (1 µM) that did not affect cell viability or proliferation (Figure 4A). The GSK126 treatment resulted in decreased H3K27me3 levels and conferred cisplatin resistance to NT2/D1, 2010EP, and 833K cells (Figure 4B,C). In a reciprocal experiment, pretreatment of randomly chosen cisplatin resistant cells NT2/D1-A4, NT2/D1-H1, 2010EP-B3, and 833K-B4 with the specific JMJD3 H3K27 demethylase inhibitor, GSKJ4, (0.5 µM) resulted in increased H3K27me3 and sensitized the cells to cisplatin (Figure 4B–D). These results indicate that modulating H3K27 methylation alters cisplatin sensitivity in TGCT cells.

### 2.4. A H3K27me3-Based Gene Signature Predicts Cisplatin Sensitivity in TGCT Cells and Disease-Free Survival in TGCT Patients

**Figure 5:**
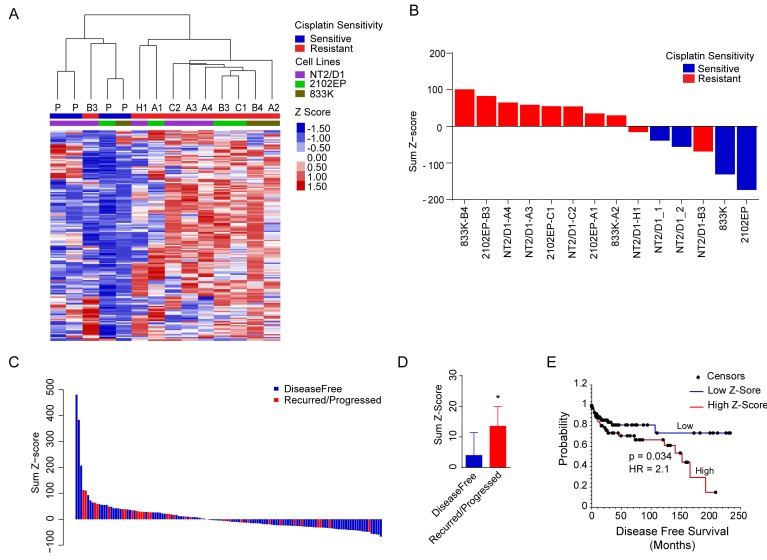
H3K27me3-based gene signature predicts cisplatin sensitivity in TGCT cells and disease-free survival in TGCT patients. A candidate gene signature was developed using enriched genes of PRC1, PRC2, and H3K27 methylation gene sets from the GSEA analysis as described in Materials and Methods. (**A**) The H3K27me3 gene signature distinguishes cisplatin sensitive and resistant cells. The heat map represents unsupervised hierarchal clustering of individual *Z*-score values for genes in the signature across the parental and resistant lines. The average of the biological triplicate values was used for clustering analysis. (**B**) The sum of *Z*-scores of signature genes in parental and cisplatin resistant cells. (**C**) Expression of the H3K27me3 gene signature is enriched in primary TGCT samples in patients that recur and progress. The waterfall plot of the sum of *Z*-scores from genes from the H3K27me3 gene signature. The blue and red bars represent disease-free and recurred patients, respectively. Gene expression data were from TCGA downloaded from http://www.cbioportal.org/. (**D**) The average sum *Z*-score from 98 disease-free and 34 recurred/progressed patients. Error bars represent SEM. * indicates *p*-value < 0.05. (**E**) High expression of H3K27me3 signature genes is associate with a decrease in disease-free survival in TGCT patients. Kaplan–Meier survival analysis was performed from the dataset described in C above with cases divided into two groups above and below the median of the sum of *Z*-scores.

We developed a H3K27me3 gene signature based on the results from our GSEA analysis. Leading edge genes from the top 20 gene sets enriched for each resistant line and related to PRC1, PRC2, and H3K27 methylation targets were extracted (Supplementary Tables S2 and S5). While each cell line showed enrichment in the expression of the polycomb targets, there was some line-to-line variability in exactly which polycomb genes were upregulated. Genes that appeared in four or more resistant cell lines were included in the gene signature which resulted in 181 genes (Supplementary Table S6. With the exception of line NT2/D1-B3, the *Z*-scores from the signature genes were able to segregate parental from resistant cell lines upon unsupervised hierarchal clustering despite being of three distinct cell line origins (Figure 5A, B). As predicted, the majority of the signature genes were upregulated in all the cisplatin resistant cell lines indicating suppression of the PRC1/2 activity and transcriptional activation of their target genes (Figure 5A).

To probe the clinical relevance of polycomb activity in the TGCTs, RNA-seq and clinical data from TCGA were downloaded from the cBioPortal website, and consisted of 65 non-seminomas, 65 seminomas, 20 embryonal carcinomas, and six not identified; as well 132 samples that were linked to clinical data. There were 175 genes with expression values from the 181 genes of the gene signature. The sum of the *Z*-scores from each gene of the 175 genes from the 132 patients was calculated. Patients that recurred or progressed on cisplatin-based therapy had a significantly higher sum *Z*-score as compared with disease-free patients (*p* = 0.04) (Figure 5C,D). When patients were divided by the median of the sum *Z*-score, Kaplan–Meier analysis indicated that those with a higher expression of the H3K27me3 gene signature had a lower rate of disease-free survival (*p* = 0.034, hazard ratio = 2.1) (Figure 5E).

## 3. Discussion

Acquired resistance to chemotherapy and targeted therapy is arguably the single most important impediment to curative treatments of advanced cancers. The TGCTs are one of the few examples of an advanced solid tumor that is cured at a high rate with standard chemotherapy. Hence, understanding mechanisms of acquired resistance in TGCTs may shed light on mechanisms that account for resistance avoidance and chemosensitivity of TGCTs, which is currently poorly understood. Having a greater understanding will benefit not only TGCT patients but may lead to novel strategies to sensitize other solid tumors to chemotherapy while avoiding resistance.

In the present study, we used de novo approaches that unexpectedly uncovered an important role for polycomb in TGCT sensitivity and resistance to cisplatin, and we are the first to implicate histone methylation in the chemosensitivity of TGCTs. A highly significant upregulation of genes normally repressed by H3K27 methylation and the polycomb repressive complex 2 (PRC2) was noted in cisplatin resistant TGCT cells regardless of their parental origin which correlated with a substantial decrease in global H3K27me3, H2AK119 ubiquitination, and expression of BMI1. Importantly, repression of H3K27 methylation conferred cisplatin resistance to parental cells while induction of H3K27 methylation resulted in an increased cisplatin sensitivity. A gene signature based on H3K27me gene enrichment predicted an increased rate of recurrent/progressive disease in testicular cancer patients.

The TGCTs are malignant counterparts to normal germ cells and may possess unique mechanisms of hypersensitivity to genotoxic stress that guard against germline transmission of deleterious mutations [19]. Alterations in traditional mechanisms of cisplatin sensitivity or resistance in other solid tumors have been generally rejected as explanations for the inherent sensitivity of TGCTs [10,11]. These include a decrease in drug uptake or an increase in drug expulsion, cisplatin inactivation due to binding to sulfur-rich proteins, deficient mismatch DNA repair system, and inhibition of p53-mediated apoptosis [20,21,22]. Our de novo RNA-seq and bioinformatic analysis did not suggest an alteration in these prior mechanisms of cisplatin resistance. It would be interesting to directly assess the extent that these prior mechanisms contribute to cisplatin resistance in our cells, and conversely, whether previously reported cisplatin resistant cells lines, including the recently reported resistant cells of Gerwing et al., share similar epigenetic resistance mechanisms as reported here [23].

Despite high cure rates, a portion of metastatic TCGTs are resistant or develop resistance to standard chemotherapy regimens [24]. Furthermore, there are patients who initially respond to therapy but undergo late relapse. The findings of our study have important clinical implications for overcoming treatment failure in TGCT patients. We previously reported that DNA methylation inhibitor therapy is one strategy to sensitize cisplatin resistance TGCTs [25].

Two major polycomb repressor complexes (PRCs) are identified, PRC1 and PRC2. PRC2 contains the core components, EZH2, SUZ12, and EED, while PRC1 is comprised of BMI1, CBX, RIN1A/B, and PCH [26]. The EZH2 catalyzes H3K27 trimethylation which is a docking site for the PRC1 complex that catalyzes monoubiquitination of H2A on K119. Both histone modifications play a crucial role in the regulation of gene expression by repressing gene expression. In solid tumors, PRC2 has mainly been associated with oncogenesis and a poor outcome, as a large number of epithelial malignancies have gain-of-function mutations or overexpression of polycomb components which has spurred the clinical development of EZH2 inhibitors [27].

However, a loss-of-function of PRC2 mutations has also been shown to occur in a subset of tumor types including malignant peripheral nerve sheath tumor (MPNST), pediatric gliomas, myelodysplastic syndrome/AML, and T cell acute lymphoblastic leukemia [28,29,30,31]. This highlights the complex role of polycomb in tumorigenesis. In contrast, very little information exists concerning the role of polycomb signaling in TGCTs. Here, we report for the first time that repression of polycomb signaling confers cisplatin resistance to TGCT cells. The finding that repression of PRC1/2 signaling induces cisplatin resistance in TGCTs was unexpected. DNA hypomethylation has been suggested to be a mechanism for TGCT cisplatin sensitivity and we and others have recently shown that pharmacologic inhibition of DNA methylation sensitizes TGCT resistant cells to cisplatin [32,33,34]. The crosstalk between DNA methylation and PCR1/2 in TGCT is likely complex and requires further study. The TCGA and other exome sequencing studies have not identified any recurrent mutations or genomic alterations in PRC1 and PRC2 genes in TGCTs.

Previous studies have linked increased PRC2-mediated epigenetic changes with cancer drug resistance. Higher expression of EZH2 or H3K27m3e was associated with cisplatin resistance in ovarian cancer, non-small cell lung cancer, osteosarcoma, and glioblastoma. Hypermethylation of H3K27 in these studies was linked to the suppression of tumor suppressor genes such as RASSF1A, MLH1, and CYT19, as well as apoptosis [35,36,37]. In contrast to these reports and in line with our findings, loss of function of histone methyltransferase EZH2 mediated multidrug resistance in AML [28,30]. Due to their pluripotent primary germ cell origins, TGCT and EC are known to have unique epigenetics states as compared with other solid tumors, including unique patterns of DNA methylation and pluripotency-associated bivalent histone modifications [14,38]. Interestingly, decreased H3K27 methylation has been previously associated with increased DNA promoter methylation in pluripotent ES cells which share many similarities with EC cells [39,40,41].

## 4. Materials and Methods

### 4.1. Derivation of Cisplatin Resistant Cells

All cells were cultured in DMEM (Gibco, Gaithersburg, MD, USA) with 10% FBS (Invitrogen, Carlsbad, CA, USA). NT2/D1, 833K, and 2102EP are human testicular cancer derived EC cell lines purchased from ATCC and authenticated by ATCC with karyotyping and short tandem repeat profiling. Cells were frozen within 1 month of purchase and used within 2 months of resuscitation. For generation of cisplatin resistant cell lines, parental cells were exposed to stepwise dosages of cisplatin (Sigma-Aldrich, St. Louis, MO, USA) starting at 0.5 µM for 3 h for 5 consecutive days and then allowed to recover for 2–3 weeks (Figure 1A). This cycle was repeated 5 times with a final selection at 10 µM cisplatin. Clones were then derived from each pool with cloning cylinders (Supplementary Table S1). Each letter designation refers to selection from an independently selected batch of cells. Lines with a common letter designation refer to lines cloned from a common pool of resistant cells (for example NT2/D1-A3 and NT2/D1-A4). All clones were stably resistant since resistance to cisplatin was retained after passaging in cisplatin-free media for at least 4 months.

### 4.2. Drug Treatments and Cell Viability and Proliferation Assays

Cells were treated with the indicated dosages of cisplatin for 6 h and cells assayed for survival 3 days later (Figure 1B). For sequential treatments cells were pretreated with the EZH2 inhibitor, GSK126, or the JMJD3 histone demethylase inhibitor, GSKJ4, (both from Selleck Chemicals, Houston, TX, USA) for 3 days at doses that alone did not affect viability by more than 5% (0.5 µM and 1.0 µM, respectively) and then treated with cisplatin. To assess cell viability, CellTiter-Glo (Promega, Madison, WI, USA) assays were performed. For each cell line, six biological replicates were tested at each concentration, and experiments were repeated at least twice on different days. The IC50 values were estimated from the best-fit dose-response model selected by residual standard error using GraphPad Prism software (GraphPad Software, San Diego, CA, USA).

To estimate cell doubling times, equal number of cells were plated in 24-well plates and viable cell numbers estimated for 5 consecutive days using the CellTiter-Glo assay. Doubling times were calculated with an exponential growth curve equation using GraphPad Prism software.

### 4.3. Cisplatin Adduct Assay

The cisplatin adduct assay was based on the protocol of Han et al. [42]. Briefly DNA was isolated from control or cisplatin treated cells and 100 ng of DNA was loaded onto nitrocellulose membranes. The level of platinum-GG (Pt-GG) adducts was determined using monoclonal anti-Pt-GG antibody (Abcam, Cambridge, MA, USA). Membranes were stripped and probed with mouse anti-single-stranded DNA (ssDNA) antibody (Millipore, Burlington, MA, USA) to serve as loading control.

### 4.4. RNA-Sequencing

RNA was extracted from indicated parental and cisplatin resistant cell lines in biological triplicate using the RNeasy Mini Kit (Qiagen, Hilden, Germany). All downstream analysis presented in Figures and Tables included the biological triplicates of each cell line. RNA sequencing was performed by the Roy J. Carver Biotechnology Center (UIUC, Champaign, IL, USA). RNA-Seq libraries were prepared using the TruSeq Stranded mRNA Sample Prep kit (Illumina, San Diego, CA, USA). The libraries were sequenced on a HiSeq 4000 using HiSeq 4000 sequencing kit version 1 (Illumina, San Diego, CA, USA). A quality control of reads generated from RNA sequencing was performed using FASTQC. Trimmomatic was used to remove the low-quality bases from starts and ends with threshold of LEADING <28 and TRAILING <28, respectively with minimum length of 30. The resulting clean reads were then aligned to human genome assembly NCBI GRCh38.p12 using STAR aligner. The resulting aligned reads from STAR aligner were used to count the reads mapping to each gene in each sample using feature counts. All the samples have comparable number of reads and comparable quality scores. The mean quality value across each base position in the read was >30 representing a 99.9% base call accuracy. Differentially expressed genes were identified by Limma Bioconductor package. To correct multiple hypothesis testing, false discovery rate (FDR) was calculated using the Benjamini–Hochberg method and genes with FDR <0.01 and absolute fold change >2 were considered differentially expressed. In all cases each cisplatin resistant cell line was compared to its corresponding parental line. The RNA-seq datasets from this study have been submitted to the NCBI Database of GEO Datasets under the accession number GSE129696.

### 4.5. Gene Set Enrichment Analysis and Gene Signature

Gene set enrichment analysis (GSEA), comparing each resistant cell line to its matching control, was performed using a maximum and minimum gene set size of 500 and 15 respectively [43]. The number of permutations were 1000 and the permutation type was set to gene set. The BART (binding analysis for regulation of transcription) tool in gene-set mode was used to predict whether differentially expressed genes were enriched for transcription factor binding sites based on more than 6000 existing ChIP-seq datasets http://faculty.vir ginia.edu/zanglab/bart [18].

A gene signature was developed using gene sets from the C2 collection from GSEA that were enriched in resistant cells as compared with their parental cells. Briefly, GSEA was performed on each cell line and genes sets enriched in the resistant cells from the C2 collection set were determined. Leading edge genes from the top 20 gene sets enriched for each resistant line and related to PRC1, PRC2, and H3K27 methylation were extracted. Genes that appeared in four or more resistant cell lines were included in the gene signature which resulted in 181 genes. The *Z*-score values for each gene across the parental and resistant lines were determined and hierarchal clustering was performed using a correlation metric for similarity and average linkage clustering. In all cases the average of biological triplicate values for each treatment group were used for clustering analysis.

### 4.6. Disease-Free Survival Analysis

Testicular germ cell cancer RNA-seq and clinical data from TCGA were downloaded from the cBioPortal website (https://www.cbioportal.org/), and consisted of primary tumors from 65 non-seminomas, 65 seminomas, 20 embryonal carcinomas, and 6 not identified samples; as well 132 samples were linked to clinical data. There were 175 genes with expression values from the 181 genes of the gene signature. The sum of the *Z*-scores from each gene for the 175 genes was determined for each sample and Kaplan–Meier log-rank tests were performed using WinSTAT with samples grouped into low or high at the *Z*-score median.

### 4.7. Western Analysis and Real-Time PCR

For Western analysis cells were lysed in radioimmune precipitation buffer. Cell lysate containing equal amounts of protein (30 μg) were resolved using SDS-PAGE, transferred to nitrocellulose membranes, and blocked with 5% non-fat dry milk. Blocked membranes were incubated overnight at 4 °C with indicated primary antibodies. Antibodies to actin (1:1000; MA1-744, Thermo Fisher Scientific, Waltham, MA, USA), histone H2A (1:1000; 8240, Cell Signaling Technology, Danvers, MA, USA), histone H3 (1:1000; ab1791, Abcam, Cambridge, MA, USA), Ubiquitin H2A-K119 (1:1000; 3240, Cell Signaling Technology), BMI1 (1:1000; 6964, Cell Signaling Technology), EZH2 (1:1000; 5246, Cell Signaling Technology) and H3K27me3 (1:1000; 9733, Cell Signaling Technology) were used. Protein expression was detected using horseradish peroxidase-conjugated secondary antibody and chemiluminescence reagent (Thermo Fisher Scientific).

Total cellular RNA was isolated using the RNeasy Mini Kit (Qiagen), and complementary DNAs (cDNAs) were synthesized from 2 µg RNA using iScript Reverse Transcription Supermix (Bio-Rad Laboratories, Hercules, CA, USA). Quantitative real-time PCR assays were performed with iTaq Universal SYBR Green Supermix (Bio-Rad Laboratories) and the QuantStudio 3 Real-time System (Thermo Fisher). EZH2 primers were 5′-GTGGAGAGATTATTTCTCAAGATG-3′ and 5′-CCGATCCAATCTGTTCTGGT-3′, BMI1 primers were 5′-CCAGGGCTTTTCAAAAATGA-3’and 5′-CCGATCCAATCTGTTCTGGT-3’ GAPDH primers were 5′-GATTCCACCCATGGCAAATT-3′ and 5′-GATGGTGATGGGATTTCCATTG-3′.

### 4.8. Statistics

All experiments with error bars were performed in biological triplicate and all experiments were repeated at least twice. Two tailed student’s *t*-tests, one-way or two-way ANOVA with post hoc Bonferroni, and log-rank test were performed, where appropriate, using GraphPad Prism v6.0 and *p*-values indicative of non-significant *p* > 0.05 and significant * *p* ≤ 0.05 were determined. Mean and standard error of mean were used to describe sample variability.

## 5. Conclusions

(1) Acquired cisplatin resistance in testicular cancer cells is associated with enhanced expression of genes normally repressed by H3K27 methylation. (2) Cisplatin resistance in testicular cancer cells is associated with decreased H3K27 methylation, H2A-K119 ubiquitination, and decreased expression of BMI1 and EZH2. (3) Inhibition of H3K27 methylation mediates cisplatin resistance and inhibition of H3K27 demethylation sensitizes testicular cancer cells to cisplatin. (4) H3K27me3-based gene signature predicts cisplatin sensitivity in TGCT cells and disease-free survival in TGCT patients.

The molecular basis of cisplatin chemosensitivity and resistance in TGCTs is still poorly understood. A greater understanding of the hypersensitivity and curability of TGCTs has the potential to inform strategies to sensitize other solid tumors to conventional chemotherapies. We employed unique cisplatin resistant cell models and de novo transcriptional profiling that unexpectedly uncovered an important role for polycomb in TGCT sensitivity and resistance to cisplatin. Our findings are the first to implicate epigenetics in the chemosensitivity of TGCTs. Furthermore, we provide evidence that repression of H3K27 methylation is a mechanism of cisplatin-acquired resistance in TGCTs and suggest that drugs targeting PRC2 function may be a viable approach to overcome treatment failure in testicular cancer patients.

## Supplementary Materials

The following are available online at https://www.mdpi.com/2072-6694/11/6/796/s1, Figure S1: Differentially expressed genes in cisplatin resistant cell lines, Figure S2: Gene set enrichment of cisplatin resistant cell lines using polycomb-related human ES cell gene sets, Figure S3: Gene set enrichment analysis of downregulated genes, Table S1: Parental cells and their respective cisplatin acquired resistant clones, Table S2: Top 20 gene sets predicted by GSEA from upregulated genes in each of the resistant cell lines, Table S3: Top 20 predicted transcription factors for genes upregulated in each individual cisplatin resistant line, Table S4: Top 50 predicted transcription factors for genes commonly upregulated in cisplatin resistant lines, Table S5: Leading edge genes from top 20 GSEA gene sets related to H3K27 methylation or Polycomb repressive complex, Table S6: Genes used for gene signature.
