# Supplementary material for: Epigenetic Remodeling through Downregulation of Polycomb Repressive Complex 2 Mediates Chemotherapy Resistance in Testicular Germ Cell Tumors
Source: Cancers (Basel). 2019 Jun 8;11(6):796. doi: 10.3390/cancers11060796 (PMC6627640; doi:10.3390/cancers11060796)
Supplement: Supplementary file 1 [file cancers-11-00796-s001.zip › cancers-505886-supplement-figures.docx]

Supplementary Materials

Epigenetic Remodeling through Downregulation of Polycomb Repressive Complex 2 Mediates Chemotherapy Resistance in Testicular Germ Cell Tumors

Ratnakar Singh, Zeeshan Fazal, Andrea K. Corbet, Emmanuel Bikorimana, Jennifer C. Rodriguez, Ema M. Khan, Khadeeja Shahid, Sarah J. Freemantle and Michael J. Spinella


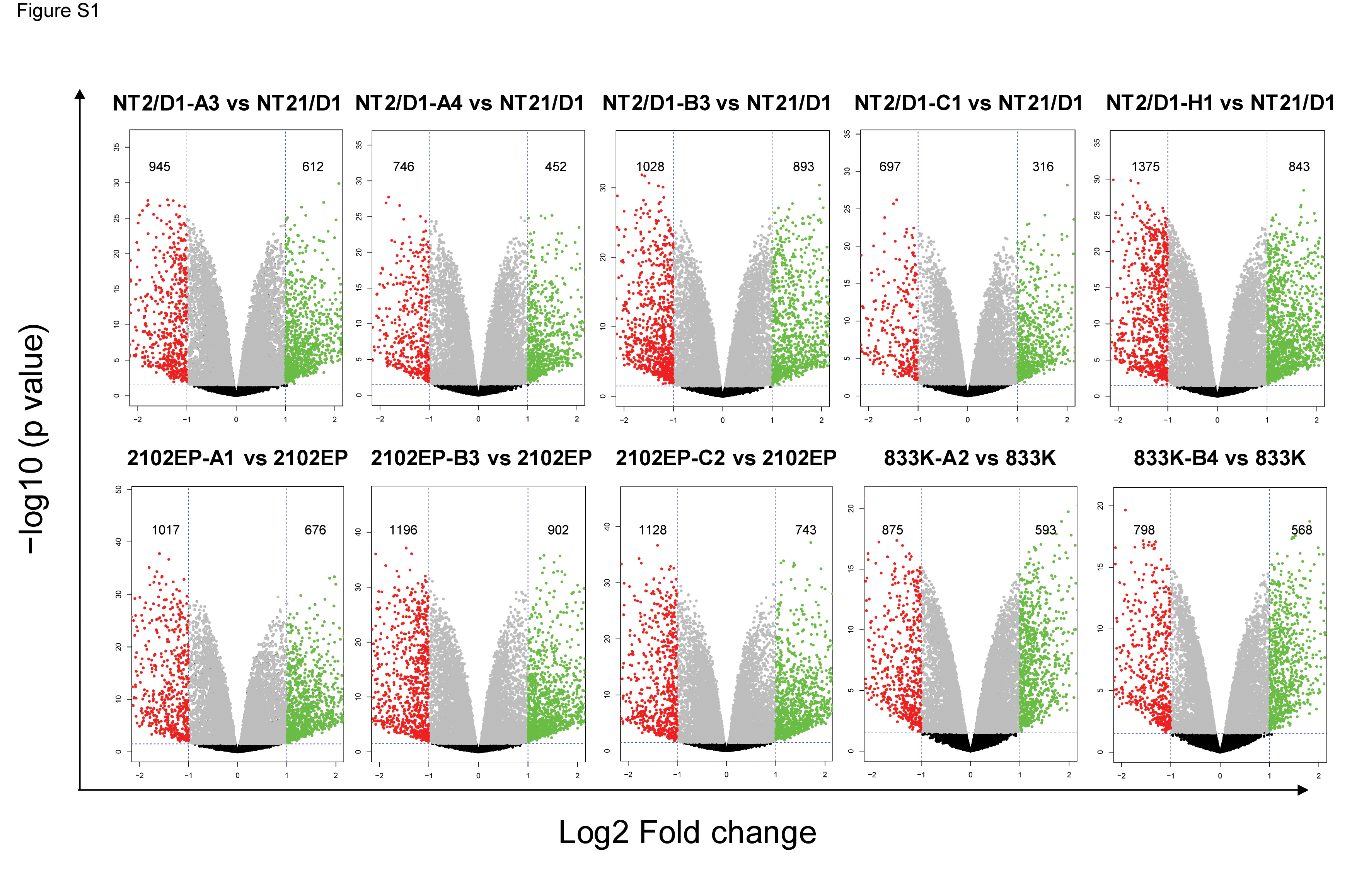


**Figure S1.** Differentially expressed genes in cisplatin resistant cell lines. Volcano plots of RNA seq data shows significantly upregulated (Green) and downregulated (Red) genes in cisplatin resistant cells lines compared to respective parental control cells. A 2-fold cut off and FDR < 0.05 was used. Average of biological triplicate determinations.


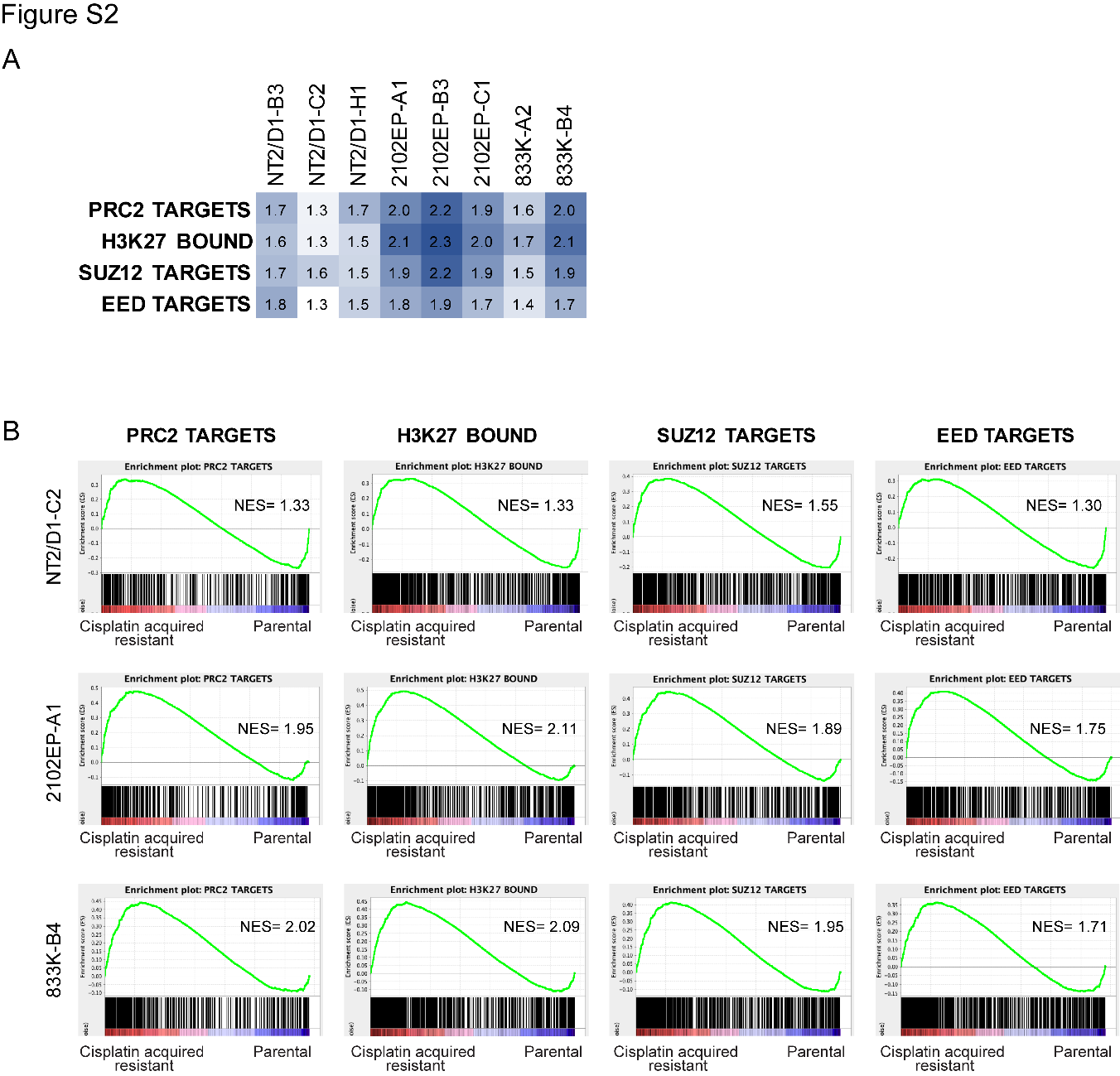


**Figure S2.** Gene set enrichment of cisplatin resistant cell lines using polycomb related human ES cell gene sets. (**A**) Normalizes enrichment scores (NES) for indicated cisplatin resistant cell lines. Value of NES is in gradation of blue. (**B**) Representative enrichment plots from **A**.


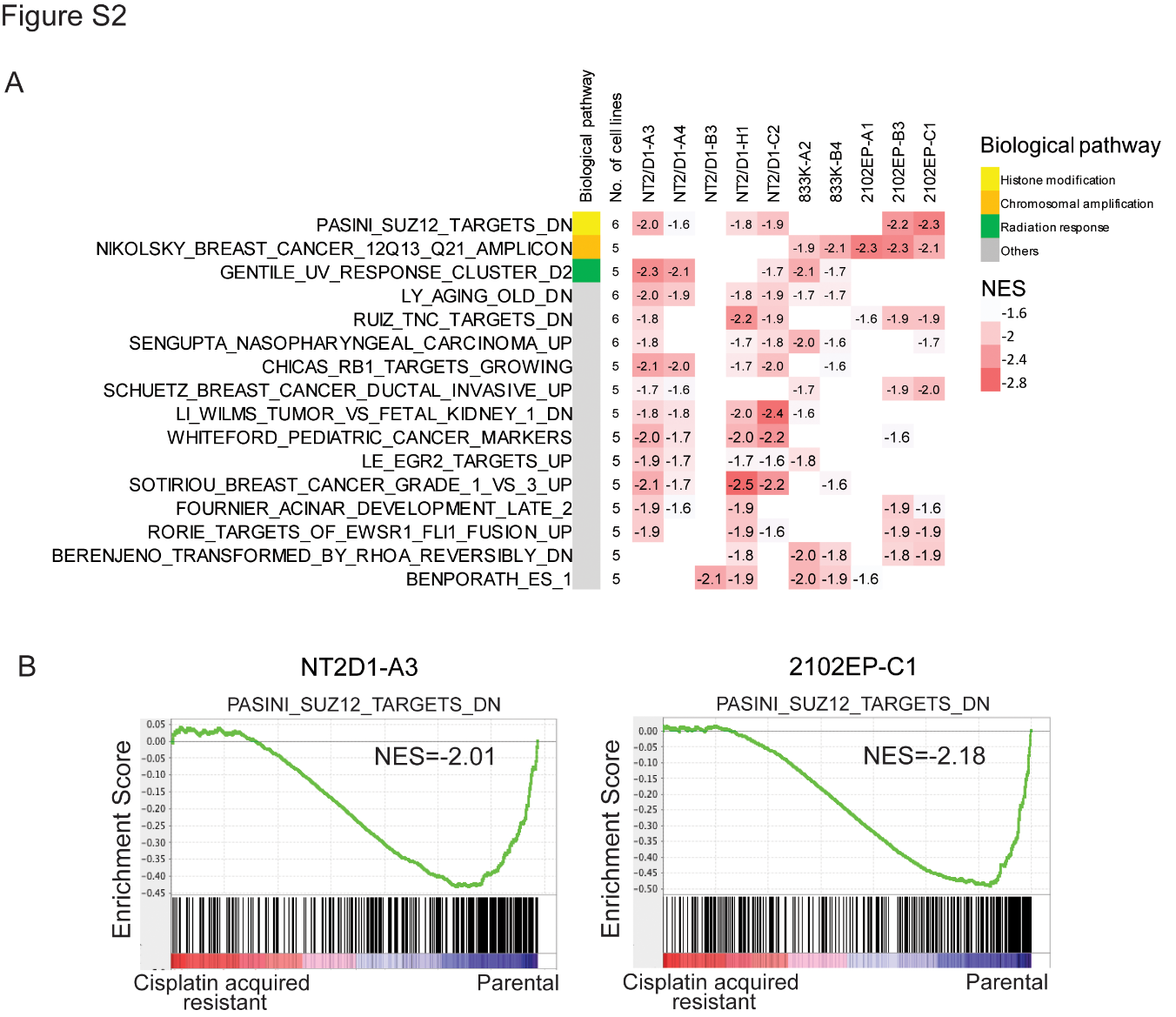


**Figure S3.** Gene set enrichment analysis of downregulated genes. (**A**) Cisplatin resistant cells are enriched for gene sets associated with PRC2 related SUZ12 targets. All gene sets that have a normalized enrichment score (NES) of −1.6 or less and are enriched in 5 or more resistant cell lines are included. Numbers and gradient of red color represent NES. (**B**) Representative gene set enrichment plots. PRC2, polycomb repressive complex 2; NES, normalized enrichment score.

*Supplementary Materials of Source Date for Figures 3B,4B (Original Western Blot Images)*


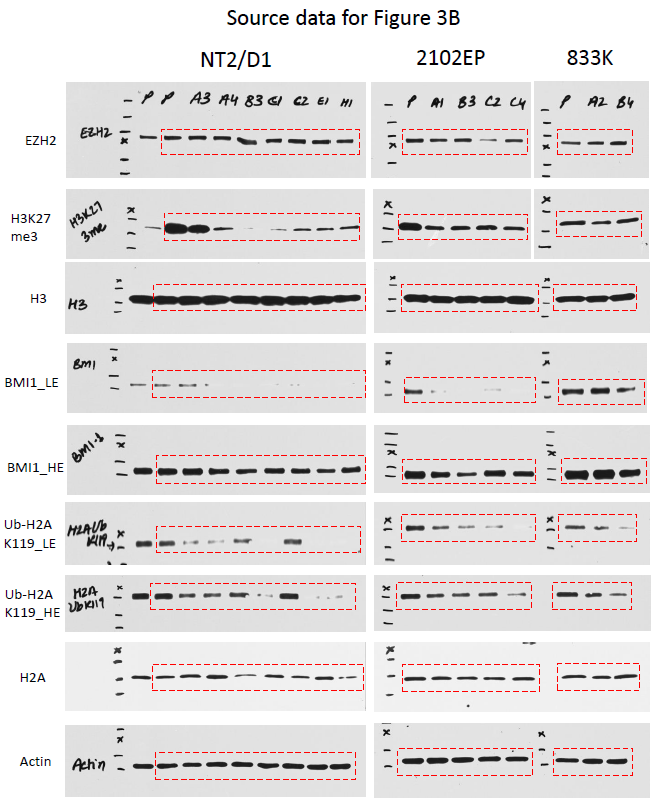


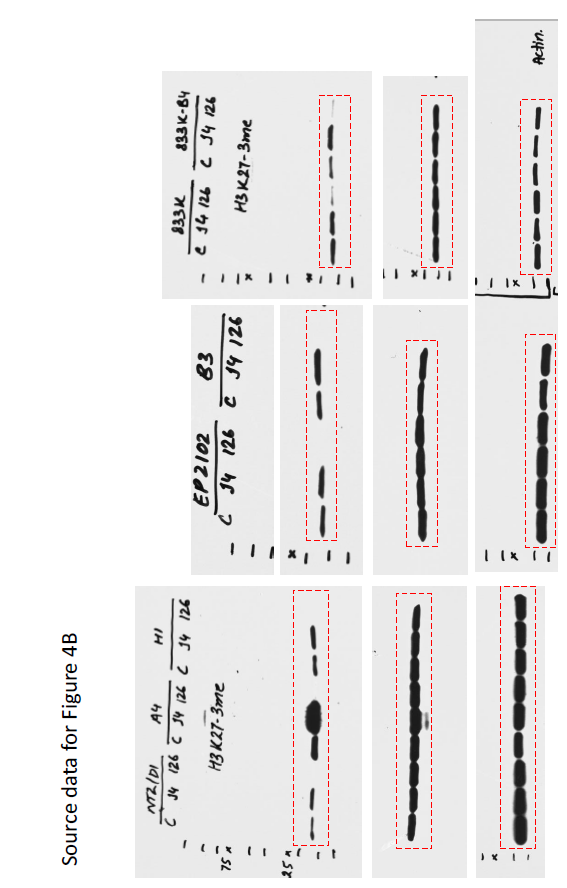


| 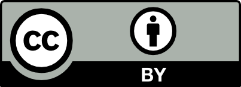 | © 2019 by the authors. Licensee MDPI, Basel, Switzerland. This article is an open access article distributed under the terms and conditions of the Creative Commons Attribution (CC BY) license (http://creativecommons.org/licenses/by/4.0/). |
| --- | --- |
